# Endocannabinoid Metabolism and Traumatic Brain Injury

**DOI:** 10.3390/cells10112979

**Published:** 2021-11-02

**Authors:** Dexiao Zhu, Fei Gao, Chu Chen

**Affiliations:** Health Science Center, Department of Cellular and Integrative Physiology, Long School of Medicine, University of Texas, San Antonio, TX 78229, USA; gaof1@uthscsa.edu (F.G.); chenc7@uthscsa.edu (C.C.)

**Keywords:** endocannabinoid, cannabinoid receptor, traumatic brain injury, Alzheimer’s disease, monoacylglycerol lipase, proliferator-activated receptor γ

## Abstract

Traumatic brain injury (TBI) represents a major cause of morbidity and disability and is a risk factor for developing neurodegenerative diseases, including Alzheimer’s disease (AD). However, no effective therapies are currently available for TBI-induced AD-like disease. Endocannabinoids are endogenous lipid mediators involved in a variety of physiological and pathological processes. The compound 2-arachidonoylglycerol (2-AG) is the most abundant endocannabinoid with profound anti-inflammatory and neuroprotective properties. This molecule is predominantly metabolized by monoacylglycerol lipase (MAGL), a key enzyme degrading about 85% of 2-AG in the brain. Studies using animal models of inflammation, AD, and TBI provide evidence that inactivation of MAGL, which augments 2-AG signaling and reduces its metabolites, exerts neuroprotective effects, suggesting that MAGL is a promising therapeutic target for neurodegenerative diseases. In this short review, we provide an overview of the inhibition of 2-AG metabolism for the alleviation of neuropathology and the improvement of synaptic and cognitive functions after TBI.

## 1. Introduction

Traumatic brain injury (TBI) is defined as a disruption of brain function caused by external forces, including falls, blows, or blasts. TBI is one of the most challenging health concerns and a major cause of trauma-related morbidity and mortality. Each year, approximately 69 million individuals suffer TBI worldwide [1]. Depending on whether the skull is broken, TBI can be classified into two types: closed-head TBI and penetrating TBI (also called open TBI). Closed-head TBI is the most common type among patients with TBI and is generally caused by a blunt impact in vehicle accidents and contact sports activities. Based on its severity, determined using the Glasgow Coma Scale (GCS) scores, TBI is also classified as mild, moderate, or severe [2]. Clinical symptoms of TBI are coma, headache, seizures, amnesia, and behavioral changes. TBI not only causes immediate tissue damages, but also induces potential long-term biochemical and neuropathological changes, including oxidative stress, excitotoxicity, disruption of blood–brain barrier (BBB) permeability, neuroinflammatory responses, and cognitive deficits [3]. Most of the symptoms happen seconds to hours following TBI, and some symptoms may persist for days, months, or years [4,5]. It has been proposed that TBI is an important risk factor for developing Alzheimer’s disease (AD), stroke, Parkinson’s disease (PD), and epilepsy [6,7,8,9,10,11,12,13,14,15].

**Figure 1:**
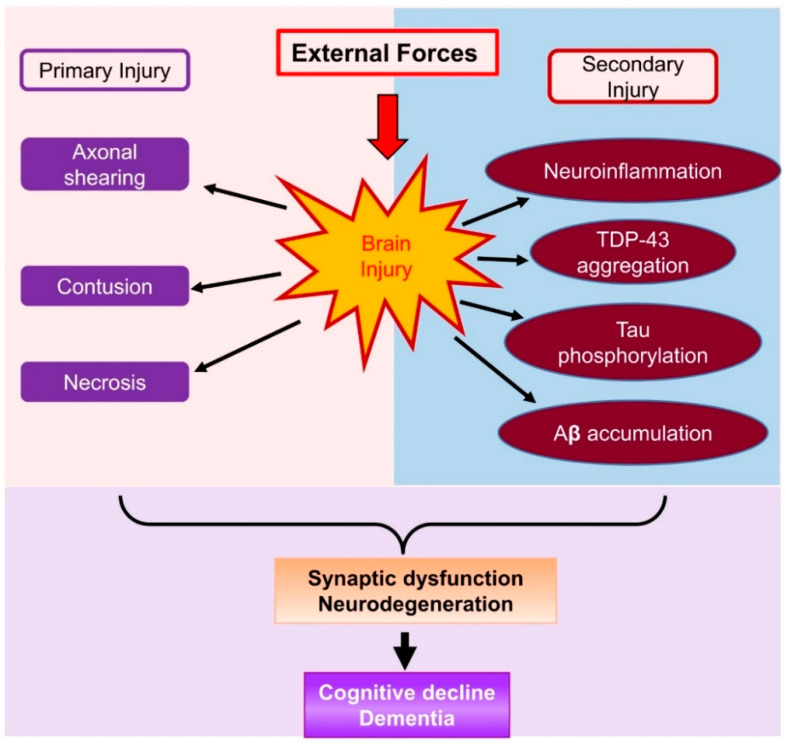
A schematic representation of brain damage following TBI, which causes primary injury and secondary injury. Primary injury occurs immediately after TBI, and secondary injury is initiated from minutes to hours following TBI. Secondary injury involves a cascade of pathophysiological processes including neuroinflammatory responses, tau phosphorylation, TDP-43 aggregation, and Aβ accumulation. These neuropathological changes following TBI lead to neurodegeneration, synaptic dysfunction, and cognitive decline.

The brain damage following TBI can be divided into primary and secondary injury (Figure 1). Primary injury results directly from the external mechanical disruption of brain tissue, occurs at the time of the insult, and is usually not alterable. Secondary injury refers to a cascade of biochemical, cellular, and pathological processes, including inflammatory response and neuronal degeneration in subcortical and deep white matter tissue [16]. The secondary injury is usually reversible and occurs within seconds or minutes following the primary damage. However, these secondary injuries can persist for minutes, days, or years. Compared to the primary injury, the secondary injury is revisable and thus provides a window for medical interventions. The inflammatory response is one of the major features of brain damage in the case of secondary injury. Neuroinflammation can induce and interact with many cellular and biochemical processes and consequently result in neuronal degeneration, phosphorylation of tau proteins, aggregation of TAR DNA-binding protein 43 (TDP-43), synaptic impairments, cognitive decline, and eventually dementia (Figure 1). Therefore, resolving neuroinflammation may prevent or delay secondary injury-induced neuropathological events. Endocannabinoids are endogenous cannabinoids with anti-inflammatory properties. In particular, 2-arachidonoylglycerol (2-AG), the most abundant endogenous cannabinoid, displays profound anti-inflammatory and neuroprotective effects both in vitro and in vivo [17,18,19,20,21,22,23]. Thus, boosting 2-AG signaling is likely an ideal approach to the resolution of neuroinflammation following TBI [24].

## 2. Endocannabinoid 2-AG Synthesis and Metabolism

**Figure 2:**
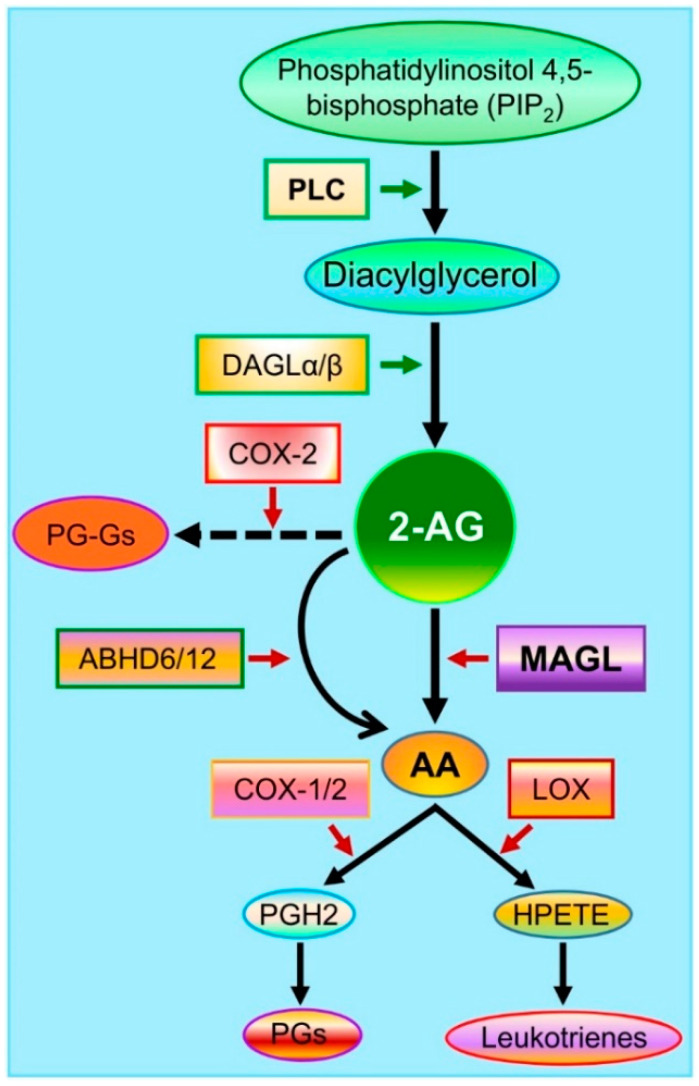
Major pathways of 2-AG synthesis and metabolism. Membrane phospholipids are converted to diacylglycerol (DAG) by phospholipase C (PLC) and then to 2-AG by diacylglycerol lipases (DAGLα and β). The compound 2-AG is hydrolyzed by the enzymes monoacylglycerol lipase (MAGL) and α/β hydrolase domain-containing proteins 6 and 12 (ABHD6/12) to glycerol and arachidonic acid (AA) and oxidatively metabolized by cyclooxygenase-2 (COX-2) to form a new type of prostaglandin glycerol esters (PG-Gs). AA is a precursor of prostaglandins (PGs) through the enzymes COX-1/2 and of hydroperoxyeicosatetraenoic acid (HPETE) through the enzyme arachidonate 5-lipoxygenase (LOX) to form leukotrienes.

The endocannabinoid 2-AG is the second identified endocannabinoid and a full agonist for cannabinoid receptors 1 and 2 (CB1 and CB2) [25,26]. While 2-AG is produced through several pathways, diacylglycerol lipases (DAGL), including DAGLα and β, are the main enzyme for the synthesis of 2-AG from diacylglycerol (DAG). Recent studies provide insights into cell type-specific synthesis of 2-AG [27,28]. For instance, 2-AG in neurons and astrocytes is primarily synthesized by DAGLα, while DAGLβ is responsible for 2-AG formation in microglial cells [27,28]. The compound 2-AG is an unstable lipid and is rapidly degraded by several enzymes, including monoacylglycerol lipase (MAGL), α/β hydrolase domain-containing proteins 6 and 12 (ABHD6/12), cyclooxygenase-2 (COX-2), cytochromes, and lipoxygenases (Figure 2). Although 2-AG can be degraded by these enzymes upon its formation, it is predominantly metabolized by MAGL, a serine hydrolase firstly purified from the rat adipose tissue in 1976 [29]. It has been estimated that 85% of 2-AG in the brain is degraded by MAGL [30,31]. MAGL plays an important role in lipid metabolism and is highly expressed in neurons and astrocytes in the brain. It is clear now that MAGL is the primary enzyme hydrolyzing 2-AG in neurons and astrocytes, while 2-AG in microglial cells is largely degraded by ABHD12 [28]. There is a 3 to 5-fold increase in brain 2-AG content in astrocytic and neuronal MAGL knockout animals, respectively [27]. However, no significant differences in the brain levels of 2-AG were observed between normal control and microglial MAGL knockout mice [27]. These studies provide important information indicating that synthesis and metabolism of 2-AG in the brain are cell type-specific, which may underlie their different functional roles in physiological and neuropathological processes.

The immediate metabolites of 2-AG are glycerol and arachidonic acid (AA, Figure 2). Arachidonic acid is a precursor of prostaglandins (PGs) through cyclooxygenase-1 (COX-1) and COX-2 and of leukotrienes (LT4s: A4 to E4) through the enzyme arachidonate 5-lipoxygenase (LOX, Figure 2). AA-derived prostaglandins and leukotrienes are proinflammatory and neurotoxic [32], while 2-AG displays anti-inflammatory and neuroprotective properties [17,18,19,20]. Besides PGs and LT4s, lipoxins (LXs), another class of derivatives from AA, display anti-inflammatory properties. LXs, including LXA4 and LXB3, are synthesized from AA through two major routes involving the cooperation of three major enzymes, including 5-LOX, 15-LOX, and 12-LOX [33,34]. Their derivatives (LXs) and aspirin-triggered LXs (ATLs) are synthesized from AA by 15-LOX and acetylated COX-2. Both LXs and ATLs can act on several types of receptors, including G protein-coupled lipoxin A4 receptor ALX/formyl peptide receptors (FPR2), aryl hydrocarbon receptors, and G protein-coupled receptor 32 [35,36,37]. From this point of view, MAGL is likely an ideal therapeutic target for neurodegenerative diseases [38,39,40].

## 3. Resolving Neuroinflammation and Maintaining the Integrity of the Blood–Brain Barrier by the Inhibition of 2-AG Metabolism in TBI

Neuroinflammation instigated by TBI is a complex immune process resulting from a mechanical insult (blast, diffuse, or focal concussion) and depending on the degree of the insult (severe, moderate or mild) and is one of the neuropathological features in TBI. Neuroinflammatory responses occur immediately following TBI and thus are an important trigger of progressive brain damage. TBI induces widespread neuroinflammation in the brain and is characterized by the entering of peripheral monocytes due to increased permeability of the blood–brain barrier (BBB), activation of resident microglia, and release of inflammatory cytokines, chemokines, and prostaglandins [41]. Microglia, developed from macrophages or monocytes, are the major resident immune cells in the brain. Quiescent microglia transform into activated cells following an external injury, and this process is mediated by the generation and release of pro- and anti-inflammatory cytokines. Experimental and clinical evidence indicate that microglia quickly transform into M1 pro-inflammatory cells immediately following TBI or M2 anti-inflammatory cells that can release trophic factors including insulin-like growth factor-1 [42,43]. Although microglia may have beneficial effects by clearing cell debris and dead cells, excessive production of pro-inflammatory cytokines produced by activated microglia appears to contribute to the pathological progression in TBI [44]. Astrocytes are also an important component of neuroinflammatory responses in TBI [45]. Interestingly, TBI-induced neuroinflammatory responses can be mitigated by the inactivation of MAGL. It has been shown that expression of cytokines (e.g., IL-1β, IL-6, TNFα), reactivity of astrocytes and microglia, and levels of nicotinamide adenine dinucleotide phosphate oxidase (NOX2) and COX-2 are significantly reduced in TBI animals treated with JZL184, a potent MAGL inhibitor [40,46,47]. Disruption of MAGL function by JZL148 prevents 2-AG degradation and raises 2-AG levels in the brain. Inactivation of MAGL, in the meantime, also reduces 2-AG metabolites (e.g., prostaglandins) [27,48]. In particular, a large proportion of prostaglandins in the brain is derived from 2-AG [48]. It is likely that enhanced 2-AG signaling and reduced PGs induced by MAGL inactivation contribute to the resolution of neuroinflammation in TBI.

The anti-inflammatory effects of LXs and ATLs in TBI appear to be via binding to FPR2 to suppress cytokines, including IL1β, IL6, and TNF, in mice [49]. While the amount of LXs might be reduced by inactivation of MAGL, the overall effects of MAGL inactivation are anti-inflammatory and neuroprotective, suggesting that enhanced 2-AG signaling by MAGL inactivation plays a dominant role in the alleviation of TBI-induced neuropathology and synaptic and cognitive deficits [24,40,50].

TBI, following even a mild head impact, could result in the breakdown of the BBB and the subsequent brain entry of peripheral immune cells and plasma protein [51]. These peripheral components can exacerbate neuroinflammation, excitotoxicity, and neurodegeneration in the brain after brain injury. Administration of exogenous 2-AG has been shown to protect the BBB in an animal model of closed-head injury and suppress TBI-induced expression of inflammatory cytokines [18,46]. Enhancement of endogenous 2-AG levels by inhibition of 2-AG metabolism with WWL70, a selective ABHD6 inhibitor, prevented BBB dysfunction following TBI, which was accompanied by attenuated neuronal degeneration, neuroinflammation, and deficits in working memory performance [52]. Maintaining the integrity of the BBB by the inhibition of 2-AG degradation was further confirmed by the inactivation of MAGL with JZL184 [46]. These animals also showed improved neurological and behavioral recovery, as well as BBB integrity. Meanwhile, TBI-activated astrocytes and microglia were also diminished in animals treated with JZL184 [46]. These studies provide evidence that inhibition of 2-AG metabolism prevents BBB dysfunction and resolves neuroinflammation following TBI, which is key to preventing secondary/further brain damage and neuropathological consequences.

## 4. Alleviation of TBI-Induced Neuropathology by Inactivation of MAGL

Hyperphosphorylated tau protein and aggregation of TDP-43 are neuropathological consequences of TBI. Hyperphosphorylated tau is one of the neuropathological hallmarks of AD and is the main component of neurofibrillary tangles (NFTs) [53,54,55]. In the nervous system, tau proteins are abundantly found in neuronal axons, but they are also expressed in somatodendritic compartments and in oligodendrocytes [56,57]. Generally, the binding of tau to microtubules is modulated by phosphorylation and dephosphorylation. However, abnormal phosphorylation leads to the release of microtubule-bound tau and the generation of NFTs. Increased tau phosphorylation has been demonstrated in models of mild and severe TBI [58]. In a recent study, Edwards et al. reported that an increase in tau accumulation was observed as early as one day after the initial injury in the cortex, amygdala, hippocampal area, and brainstem, with robust deposition on the ipsilateral side of the impact [59]. The robust deposition of tau on the contralateral side of the brain appeared one week later. This suggests TBI as a risk factor for tauopathies through the induction of tau hyperphosphorylation and aggregation. Hyperphosphorylated tau protein promotes NFTs accumulation in axons, resulting in impaired synaptic activity and induction of cells death, which may exacerbate the secondary injury post TBI. For example, C57Bl/6J mice inoculated with brain homogenates from TBI mice showed memory deficits and widespread phosphorylated tau throughout the brain 4, 8, and 12 months after inoculation [60]. Significant synaptic loss and reduction in postsynaptic density in the hippocampus were also observed after inoculation of TBI-brain homogenates [60]. Therefore, preventing or limiting tau phosphorylation may promote recovery from TBI. A previous study demonstrated that pharmacological inhibition of MAGL reduced the levels of phosphorylated tau as well as of P25 and phosphorylated GSK3β, key players in tau phosphorylation, 8 and 30 days after the first injury in a mouse model of repetitive mild closed-head injury [40], suggesting that inhibition of 2-AG metabolism is capable of suppressing tau phosphorylation. A later study in a tau animal model of AD supports this notion. The authors showed that JZL184 significantly reduced the levels of phosphorylated GSK3β and phosphorylated tau, including p-tauT181 and p-tau (Ser202, Thr205), and improved spatial learning and memory retention in the animals [39].

Transactivation response DNA-binding protein 43 (TDP-43), which is expressed in most tissues, regulates transcription and exon splicing via binding to both DNA and RNA. In amyotrophic lateral sclerosis (ALS), TDP-43 is hyperphosphorylated, ubiquitinated, cleaved into fragments (25 and 35 kDa) and mislocalized in the cytoplasm of neurons and glial cells [61]. TDP-43 has been proved to be linked to amyotrophic lateral sclerosis (ALS) and frontotemporal lobar degeneration (FTLD) [61]. It is also identified as an important characteristic feature in several neurodegenerative diseases, including AD and PD [62,63,64]. Studies show that TDP-43 is a crucial disease-associated protein in repetitive or concussive TBI. Increased TDP-43 breakdown fragments (35, 33, and 12 kDa) and redistributed TDP-43 from the nucleus to the cytoplasm are observed in TBI models [65,66]. Increased levels of TDP-43 and its 35 kDa fragment are also present in the cerebrospinal fluid (CSF) of severe TBI patients [67]. A clinical study reported that widespread TDP-43 accumulation occurred in patients with chronic traumatic encephalopathy (CTE), a TBI-triggered neurodegenerative disease [66]. TDP-43 abnormality induced by TBI worsened the brain injury. For instance, TBI aggravated cell death, TDP-43 abnormality, and cognitive impairments in TDP-43A315T mice [65]. Our previous study also found that expression of TDP-43 was persistently increased in the cortex and hippocampus in a mouse model of repetitive mild closed-head injury [40]. Importantly, this study revealed that pharmacological inactivation of MAGL robustly reduced TDP-43 production, providing the first evidence that inhibition of 2-AG metabolism prevents TBI-induced excessive formation of TDP-43, which, in turn, promotes recovery from the secondary injury, thus preventing cognitive decline [40].

Aβ peptides are peptides constituted by 36–43 amino acids and the main component of the amyloid plaques in the brain of patients with AD. Several lines of evidence from preclinical and clinical studies indicate that accumulation of Aβ peptides occurs within hours after brain injury and that Aβ is spread throughout the cerebral cortex [68]. Expression of APP, β-secretase, and nicstrin (NCT, a component of γ-secretase), as well as formation of Aβ are significantly increased in a mouse model of repetitive mild closed-head injury [40]. Rapid co-accumulation of APP with its cleavage enzymes (β-secretase and Presenilin-1) and Aβ production also occur in patients dying within weeks after brain trauma [69]. The produced Aβ is aggregated into plaques/oligomers, which causes apoptotic cell death, chronic inflammation, and cognitive impairments. Thus, suppression of the accumulation and deposition of Aβ will attenuate TBI-induced AD-like neuropathological changes. Our previous study showed that inhibition of 2-AG metabolism by pharmacological inactivation of MAGL repressed TBI-increased expression of APP, β-secretase, and NCT [40]. Therefore, TBI-induced neuroinflammation, TDP-43 production, tau phosphorylation, and Aβ formation, which are major neuropathological features, can be mitigated by the inactivation of MAGL, suggesting that multiple signaling pathways are involved in the protective effects produced by the inhibition of 2-AG metabolism in TBI.

## 5. Improvement of Synaptic and Cognitive Functions by Inactivation of MAGL in TBI

Long-term synaptic plasticity in terms of long-term potentiation (LTP) is a biological process referring to the ability of synapses to persistently strengthen synaptic transmission, which may underlie learning and memory [70]. Studies have revealed that TBI impairs long-term synaptic plasticity. We observed that basal synaptic transmission in terms of input–output function and LTP were impaired at CA3–CA1 synapses 30 days after repetitive mild closed-head injury [40]. The results from other studies showed that TBI significant decreased the threshold and the amplitude of population spikes as well as the amplitude of EPSPs in the hippocampal CA1 region [71]. In addition, TBI robustly suppressed post-tetanic potentiation (PTP), paired pulse ratio (PPR), and short-term plasticity in a blast-induced traumatic brain injury (bTBI) mouse model [72]. TBI-induced impairments of short- and long-term synaptic plasticity are likely associated with decreases in the expression and function of glutamate receptors. We observed that the expression of glutamate receptor subunits, including AMPA receptor subunits GluA1 and GluA2 and NMDA receptor subunits GluN2A and GluN2B, was significantly downregulated 8 and 30 days after TBI [40]. Interestingly, pharmacological inhibition of MAGL was capable of restoring TBI-reduced expression of these glutamate receptor subunits and improving basal synaptic transmission and LTP [40]. Moreover, pharmacological inhibition of MAGL decreased TBI-induced synaptic hyperexcitability in layer 5 neurons 10 days after injury [47]. Inhibition of 2-AG metabolism also attenuated TBI-induced increases in the frequency and amplitude of miniature excitatory postsynaptic currents (mEPSCs) in layer 5 pyramidal neurons of rats [73]. These studies provide important information that the inhibition of 2-AG metabolism prevents TBI-caused disturbance of brain homeostasis and synaptic dysfunction.

Neurocognitive decline and dementia are the major consequences of TBI [3,74,75,76]. Assessment of learning and memory is widely used in animal studies to evaluate cognitive functions following TBI. Since inactivation of MAGL ameliorates TBI-induced neuropathology, maintains the integrity of synapses, and improves long-term synaptic plasticity, it is likely that inhibition of 2-AG metabolism would prevent TBI-induced cognitive decline. Our study provides evidence that TBI-induced deficits in spatial learning and memory are attenuated by pharmacological inactivation of MAGL [40], suggesting that limiting 2-AG degradation following TBI is a promising approach for preventing or diminishing neuropathological and neurocognitive sequelae.

## 6. Potential Mechanisms Underlying the Neuroprotective Effects of MAGL Inactivation in TBI

**Figure 3:**
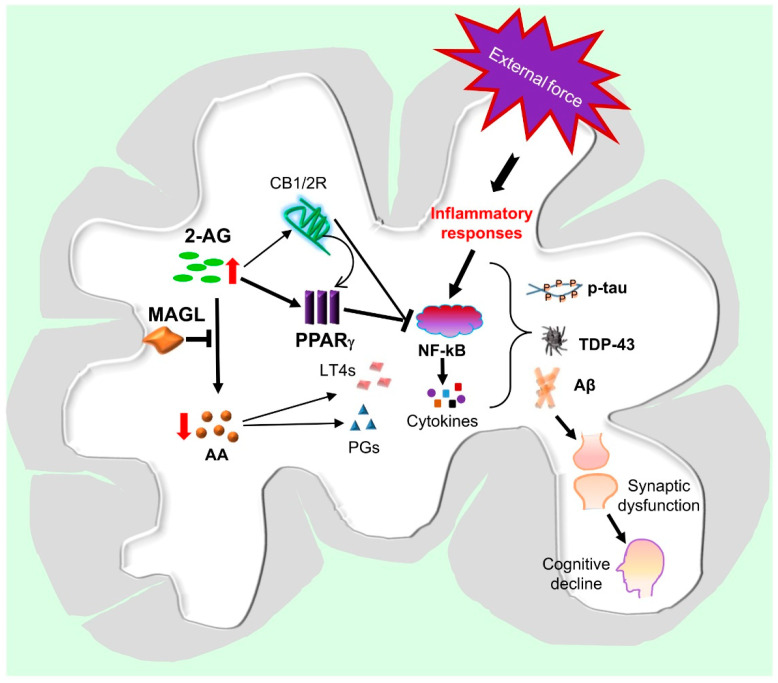
Hypothetic signaling pathways mediating neuroprotection produced by MAGL inactivation in TBI. An external force triggers the activation of inflammatory responses through NF-kB signaling in brain immune cells, including astrocytes and microglia, resulting in the release of chemokines, cytokines, and eicosanoids. These inflammatory factors promote tau phosphorylation, TDP-43 aggregation, and Aβ formation, leading to synaptic dysfunction and neurodegeneration, which, in turn, cause cognitive deficits and eventually lead to dementia. Inhibition of 2-AG metabolism by inactivation of MAGL augments the anti-inflammatory and neuroprotective 2-AG signaling, which stimulates the expression and activity of PPARγ through CB1/2-dependent and-independent mechanisms. PPARγ interacts with NF-kB to inhibit its transcriptional activity, resulting in decreases in the expression of genes involved in inflammatory and neurodegenerative processes. Inactivation of MAGL also reduces 2-AG metabolites, including arachidonic acid (AA), prostaglandins (PGs), and leukotrienes (LT4s), which are proinflammatory and neurotoxic. Resolution of neuroinflammation by inactivation of MAGL is likely a key to mitigate TBI-induced neuropathology and to improve synaptic and cognitive function.

Previous studies provided evidence that inhibition of 2-AG metabolism produced neuroprotective effects in several animal models of neurodegenerative disease [38,39,40,48,77,78,79]. However, the molecular mechanisms responsible for these neuroprotective effects are still not clear. Since CB1 and CB2 receptors are the targets of 2-AG, it is likely that these receptors may play a role in the neuroprotective effects of MAGL inactivation (Figure 3). It has been demonstrated previously that 2-AG or MAGL inhibitors, including URB602 and JZL184, exert neuroprotective effects against cytokine- or Aβ-induced insults in primary cultured hippocampal neurons and the effects appear to be mediated by CB1 receptor-dependent suppression of COX-2, ERK1/2, and NF-κB [21,80]. Inconsistent with the results of these studies, there are reports of CB1 or CB2 receptor-independent neuroprotective effects by the inhibition of MAGL. Pharmacological or genetic inactivation of MAGL reduced LPS-induced inflammatory cytokines and protected neurons from degeneration in an animal model of Parkinson’s disease in the presence of CB1 or CB2 receptor blockade or genetic deletion [48]. Similarly, pharmacological or genetic inactivation of MAGL reduced the levels of eicosanoids, Aβ, and inflammatory cytokines in the brain of PS1/APP, mice and the effects were not affected by antagonism of CB1 or CB2 receptors [78]. It has been proposed that the anti-inflammatory and neuroprotective effects of MAGL inhibition are primarily mediated by a reduction of 2-AG metabolites (AA and prostaglandins), rather than by an enhancement of endocannabinoid signaling [48,78]. Another study also revealed that JZL184 reduced the expression of APP, β-secretase, and total Aβ and Aβ42, as well as neuroinflammation in APP transgenic mice lacking CB2R [79]. The results from previous studies suggest that the mechanisms involved in the anti-neuroinflammatory and neuroprotective effects of MAGL inactivation are complex, and additional signaling pathways may also contribute to the neuroprotective effects produced by 2-AG metabolism inhibition [50].

Earlier studies revealed that administration of 2-AG produced neuroprotective effects in an animal model of closed-head injury, and the effects were mediated by CB1 receptors [17,19]. Other studies also showed that neuroinflammation, neurodegeneration, and neurotoxicity induced by cytokines, Aβ, or glutamate were attenuated by the application of 2-AG or MAGL inhibitors [21,22,23]. This indicates that 2-AG is an important signaling mediator protecting neurons against harmful insults. However, very few studies have been conducted to explore the downstream signaling pathways of 2-AG in mediating these neuroprotective effects. Peroxisome proliferator-activated receptor γ (PPARγ), a member of the nuclear receptor family functioning as transcription factor, has been proposed as a target of endocannabinoids [22,81]. An early study showed that 2-AG-induced suppression of IL-2 was not mediated through CB1R, but through PPARγ signaling in T cells, suggesting that 2-AG can directly activate PPARγ [82]. In cultured hippocampal neurons, 2-AG- or JZL184-induced CB1R dependent anti-inflammatory and neuroprotective effects were suppressed by a PPARγ antagonist, and the protective effects were mimicked by a PPARγ agonist [22]. Importantly, it was reported that PPARγ is involved in the reduction of Aβ and neuroinflammation and the improvement of spatial learning and memory induced by MAGL inhibition in a mouse model of AD [83]. The PPARγ-mediated anti-inflammatory and neuroprotective effects occur likely through suppression of NF-kB transcriptional activity [22,83,84]. In addition, activation of PPARγ can ameliorate several aspects of neuropathology following TBI. For instance, pioglitazone, a PPARγ ligand, inhibited the inflammatory response and attenuated the cognitive dysfunction associated with TBI [85]. These studies suggest that PPARγ is likely an important downstream molecule in mediating anti-inflammatory and neuroprotective effects of 2-AG signaling against harmful insults (Figure 3) [50].

## 7. Outlook on Potential Treatment Strategies for TBI

There are several MAGL inhibitors currently available. They can be classified into two main categories, i.e., irreversible (JZL184, JW651, and ABX-1431) [86,87,88] and reversible inhibitors (pristimerin and euphol) [89]. Reversible inhibitors bind to the enzyme to form a complex in a reversible way, while irreversible inhibitors bind tightly to the enzyme and persistently inactivate it, thus producing longer effects. Both irreversible and reversible MAGL inhibitors are capable of boosting 2-AG levels by suppressing the catabolic activity of MAGL and have been shown to produce anti-inflammatory effects in several animal models of neurodegenerative diseases [17,21,23]. Previous studies demonstrated that JZL184, an irreversible MAGL inhibitor, induced antagonism of the endocannabinoid system and desensitization of the CB1 receptor [90]. However, reversible MAGL inhibitors are less likely to desensitize the CB1 receptor [91,92] due to their rapid dissociation from the enzyme. Several recent studies reported that pristimerin, a reversible MAGL inhibitor, suppressed inflammatory responses both in vivo and in vitro [93,94,95,96]. Therefore, although both irreversible and reversible MAGL inhibitors would alleviate or attenuate the symptoms of TBI and TBI-induced AD-like neuropathology by resolving neuroinflammation, reversible MAGL inhibitors might provide a better therapeutic effect. Current available MAGL inhibitors are mostly irreversible. It is imperative to identify and develop novel reversible MAGL inhibitors.

## 8. Summary

In this review, we discussed the beneficial effects of the inhibition of 2-AG metabolism in TBI-induced AD-like neuropathology. We focused on MAGL, as it is the key enzyme hydrolyzing 2-AG in the brain. Apparently, both enhanced 2-AG signaling and reduction of its metabolites by inactivation of MAGL contribute to anti-inflammatory and neuroprotective effects in the context of TBI, suggesting that MAGL is likely a therapeutic target for TBI [40,50]. However, the mechanisms involved in the mitigation of neuropathology and the prevention of synaptic and cognitive declines induced by MAGL in TBI remain to be studied. Neuroinflammation is a crucial factor triggering a series of neuropathological changes, including tau phosphorylation, TDP-43 aggregation, and Aβ production, following TBI, and suppression of neuroinflammation by inhibition of 2-AG metabolism is a key in preventing TBI-caused neuropathological changes. Therefore, understanding of how TBI-triggered neuroinflammation is resolved by the inactivation of MAGL will provide a better therapeutic strategy for TBI.
